# The Role of Glucose Metabolism and Glucose-Associated Signalling in Cancer

**Published:** 2008-01-18

**Authors:** Rainer Wittig, Johannes F. Coy

**Affiliations:** 1 R-Biopharm AG, Landwehrstrasse 54, 64293 Darmstadt, Germany; 2 TAVARTIS GmbH, Kroetengasse 10, 64853 Otzberg, Germany; 3 Dept. Of Gynaecology, University of Würzburg, Josef Schneider Str. 4, 97080 Würzburg, Germany

**Keywords:** Warburg effect, glucose metabolism, cancer, TKTL1 transketolase, pentose phosphate pathway, western diet

## Abstract

Aggressive carcinomas ferment glucose to lactate even in the presence of oxygen. This particular metabolism, termed aerobic glycolysis, the glycolytic phenotype, or the Warburg effect, was discovered by Nobel laureate Otto Warburg in the 1920s. Since these times, controversial discussions about the relevance of the fermentation of glucose by tumours took place; however, a majority of cancer researchers considered the Warburg effect as a non-causative epiphenomenon. Recent research demonstrated, that several common oncogenic events favour the expression of the glycolytic phenotype. Moreover, a suppression of the phenotypic features by either substrate limitation, pharmacological intervention, or genetic manipulation was found to mediate potent tumour-suppressive effects. The discovery of the transketolase-like 1 (TKTL1) enzyme in aggressive cancers may deliver a missing link in the interpretation of the Warburg effect. TKTL1-activity could be the basis for a rapid fermentation of glucose in aggressive carcinoma cells via the pentose phosphate pathway, which leads to matrix acidification, invasive growth, and ultimately metastasis. TKTL1 expression in certain non-cancerous tissues correlates with aerobic formation of lactate and rapid fermentation of glucose, which may be required for the prevention of advanced glycation end products and the suppression of reactive oxygen species. There is evidence, that the activity of this enzyme and the Warburg effect can be both protective or destructive for the organism. These results place glucose metabolism to the centre of pathogenesis of several civilisation related diseases and raise concerns about the high glycaemic index of various food components commonly consumed in western diets.

## Introduction

Constitutive activation of signalling pathways by oncogenic events or loss of (tumour) suppressive functions is considered to be causative for tumourigenic conversion as well as tumour progression. Progress at the technological level allowed researchers to delineate cancer relevant signalling as well as to discover both connections and dependencies between dominant growth/survival pathways and their negatively regulating counterparts. As foreseen by Hanahan and Weinberg in their frequently cited review article “the hallmarks of cancer” (2000), research tends to move away from single-event analysis towards more complex analyses, culminating in the advent of systems biology (Kirschner, 2005). This yet poorly defined biological discipline is still in its infancies, but the trend already revealed that in most cases understanding cancer cannot result from the view on a single oncogenic event, but must consider the combined action of both extracellular and intracellular triggers in a given cellular background. As a further layer of complexity, cancer cells are genetically instable, which means that they actively respond to both types of signals by somatic evolution and in most cases ultimately develop an aggressive phenotype. The latter is characterized by certain almost invariant properties, which include those described by Hanahan and Weinberg (self-sufficiency in growth signals, insensitivity to antigrowth signals, evasion from apoptosis, limitless replicative potential, sustained angiogenesis, and tissue invasion and metastasis; Hanahan and Weinberg, 2000) as well as an energetically ineffective metabolism which mainly relies on the fermentation of glucose (Garber, 2004). Thus, in a more philosophical way, cancer cells may be seen as autonomous parasites within the body, which have lost the ability to fulfil their job in a given tissue, but instead develop strategies to gain independence (Pfeiffer et al. 2001).

**Figure 1.**
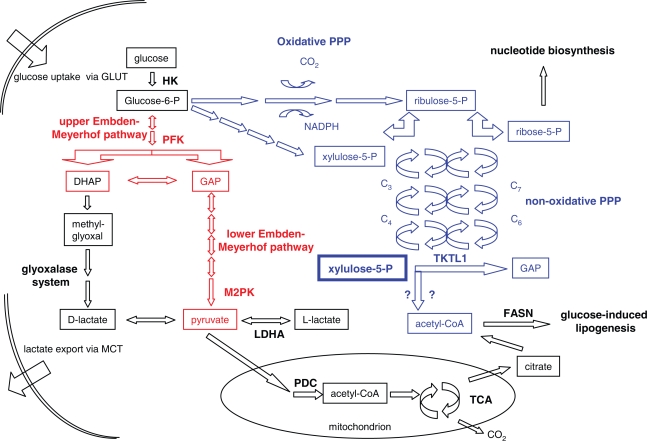
Schematic illustration of different pathways of glucose metabolism in cancer cells. Glucose enters the cell via glucose transporter proteins (GLUT). Metabolic flux rates in different pathways are determined by the regulation of rate-limiting enzymes as described in the text, as well as by the balanced equilibrium of respective intermediates. Enzymes, reactions, and intermediates of the Embden-Meyerhof pathway are coloured in red, whereas those of the PPP are coloured in blue. Selected enzymes which are referred to in the text are listed at their respective positions. The oxidative branch of the PPP delivers ribose-5-phosphate for the biosynthesis of nucleotides, as well as NADPH for reductive biosynthesis, for the regulation of the redox state within the cell, and for the detoxification of ROS. Within the illustration of the non-oxidative PPP, C_3_, C_7_, C_6_, and C_4_ indicate sugar phosphate intermediates, which are interconverted in equilibrium reactions by the transketolase and transaldolase enzymes. Xylulose-5-phosphate, the concentration of which determines the flux rates through both major pathways, may be generated from glucose-6-phosphate via the oxidative or the non-oxidative branch of the PPP. It may serve as a substrate for TKTL1 in a putative cleavage reaction, which generates GAP and a 2-carbon unit, probably acetyl-CoA. Cytosolic acetyl-CoA is utilized for glucose-induced lipogenesis. GAP, which is also generated in the Embden-Meyerhof pathway, may be further metabolized either in the Embden-Meyerhof pathway, or via methylglyoxal to D-lactate. In cancer cells, the generation of L-lactate from pyruvate is predominantly catalyzed by LDHA. Both lactate stereo-isomers are in an equilibrium with pyruvate. Since the oxidation of pyruvate is frequently inhibited in cancer cells (see text), high amounts of lactate are exported via monocarboxylate transporters (MCT).

In 1861, Louis Pasteur recognized that aerobic respiration (oxidative phosphorylation, OXPHOS) in yeast is suppressed by anaerobiosis, resulting in fermentative metabolism and the utilization of elevated amounts of glucose. Vice versa, the presence of oxygen suppresses glycolysis (the Pasteur effect). Roughly 60 years later, Nobel laureate Otto Warburg described the surprising phenomenon of elevated “aerobic glycolysis”, i.e. a fermentative glucose metabolism in the presence of oxygen, in cancerous tissues as well as in certain healthy tissues (Warburg et al. 1924). Decades later glycolysis via the Embden-Meyerhof pathway (Fig. 1) has been identified, and this way of glucose degradation has also been named glycolysis. However, as discussed in this review, the underlying molecular and biochemical bases of these two types of glycolysis are probably not identical. Therefore, the use of a single term for two different ways of glucose cleavage may have led to fatal misinterpretations of metabolic changes in tumours. In his original work, Warburg analysed the ratio of OXPHOS to glycolysis in different tissues and cancer cells and documented a particular metabolism in cancer and certain other tissues. Glycolysis under aerobic conditions was particularly high in aggressive cancers when compared to benign carcinomas and normal tissues. Elevated values for aerobic glycolysis were also found in testicular and retinal tissue. Warburg found a suppression of respiration in embryonic tissue and elevated glycolysis following exposure to cyanide and molecular nitrogen. Nitrogen evoked irreversible suppression of respiration. Based on his findings, Warburg proposed respiration deficiency and an increase of glucose fermentation as a primary cause for cancer (Warburg et al. 1924; Warburg, 1956). This interpretation was shared by another Nobel laureate, Albert Szent-Györgyi, who postulated that cancer results from a higher degree of cellular disorder, a relapse to the obligate proliferative (anaerobic) alpha state of life (Szent-Györgyi, 1980). Therefore both Nobel laureates postulated that cancer is the result of a switch from an oxygen based energy production to an anaerobic, fermentative energy production.

For decades, Warburgs hypothesis was subject of severe discussion. Subsequent to advances in molecular biology and genetics, research primarily focused on the characterization of common genetic aberrations in cancer, whereas aerobic glycolysis was frequently considered to be a non causative epiphenomenon. However, several of these genetic insults and the resulting alterations in cellular signalling have been found to be intimately linked to progressive loss of respiration as well as elevated glycolysis in carcinoma cells. Strikingly, glucose fermentation is linked to aggressiveness in cancers independent of the origin and is diagnostically exploited by the utilization of [^18^F] fluoro-2-deoxyglucose positron emission tomography (FDG-PET) (Kelloff et al. 2005). Also, inhibitors of carbohydrate metabolism were found to exhibit profound anti-tumourigenic effects (Rais et al. 1999; Du et al. 2004; Zhang et al. 2006; Ramos-Montoya et al. 2006). Therefore, during recent years tumour bioenergetics returned back into the focus of cancer research, and now prominent researchers consider aerobic glycolysis as an additional hallmark of cancer (reviewed in Garber, 2006).

## Oncogenic Signalling and the Glycolytic Phenotype of Cancer Cells

The metabolism of aggressive cancer cells is frequently dominated by a consumption of large amounts of glucose, which exceeds the needs of normal cells about 20–30 fold. Metabolic profiling experiments utilizing labelled substrate revealed, that carbon atoms of glucose predominantly appear in lactate, fatty acids, and in nucleic acid-associated ribose (Boros et al. 2002). This distribution reflects both the high proliferation rate as well as the reduction of OXPHOS in aggressive cancer cells. Glucose may be fermented by the Embden-Meyerhof glycolytic pathway (Fig. 1), which includes different rate-limiting and well regulated reactions. As described in this section, oncogenic signalling was found to interfere with the regulation of this pathway. The pentose phosphate pathway (PPP) represents an alternative route for anaerobic glucose degradation (Fig. 1), which will be discussed in more detail in the chapters below. Since it is not yet clear, how glucose is fermented in aggressive cancer cells (Coy et al. 2005), within this paper the term “glycolysis” may be solely seen as the degradation of glucose, irrespective of the enzymes involved.

In an elegant experiment, Ramanathan et al. (2005) analyzed metabolic alterations within a cellular system for cancer initiation and progression, which was modelled by the serial integration of oncogenes. Metabolic profiling revealed a progressive loss of respiration and an accompanying dependence on glycolysis for cell growth. The final introduction of the ras oncogene, which reflects a genetic aberration commonly observed in a variety of tumours, ultimately boosted resistance to oligomycin, an inhibitor of OXPHOS in these cells. The enhanced dependency on glycolysis was documented by an elevated sensitivity to 2-deoxyglucose, a potent inhibitor of glycolysis. The authors concluded that the two models of carcinogenesis, i.e. the Warburg hypothesis and the model based on cancer causing genes, are interlinked rather than being opposing models (Ramanathan et al. 2005). As described below, additional complex interactions between major oncogenic pathways determine the metabolic fate of a cancer cell, and most of the mechanisms, which lead to the phenotypic expression of the commonly accepted hallmarks of cancer (Hanahan and Weinberg, 2000) also favour the realization of the glycolytic phenotype in cancer cells.

The contribution of oncogenic ras to the glycolytic phenotype was recognized more than 20 years ago (Racker et al. 1985). More recently, detailed mechanisms leading to elevated glycolysis through ras signalling in cancer cells have been elucidated. Mazurek et al. (2001) found elevated levels of the glycolytic intermediate fructose 1,6-bisphosphate (FBP) in ras-transformed cells, which was accompanied by tetramerization and enhanced activity of the pyruvate kinase M2 isoenzyme. In another study, pharmacologic inhibition of ras signalling was found to provoke shutdown of glycolysis and subsequent cell death in glioblastoma cells (Blum et al. 2005).

Another well known key player in oncogenesis, the c-myc transcription factor, was demonstrated to transactivate the lactate dehydrogenase A (LDHA) gene, leading to an elevated production of lactic acid from pyruvate (Shim et al. 1997). The relevance of LDHA function for tumourigenicity was recently demonstrated in an RNAi-approach. Repression of LDHA stimulated OXPHOS and compromised the ability to proliferate during hypoxia (Fantin et al. 2006). C-myc-transformed cells were found to undergo apoptosis upon glucose deprivation (Shim et al. 1998). Besides LDHA, c-myc was found to transactivate a variety of other genes necessary for the glycolytic phenotype (Osthus et al. 2000). Recently, c-myc was demonstrated to initiate transcription of vascular endothelial growth factor (VEGF, an inducer of angiogenesis) in response to hypoxia and PI3K signalling (Mizukami et al. 2006).

**Figure 2.**
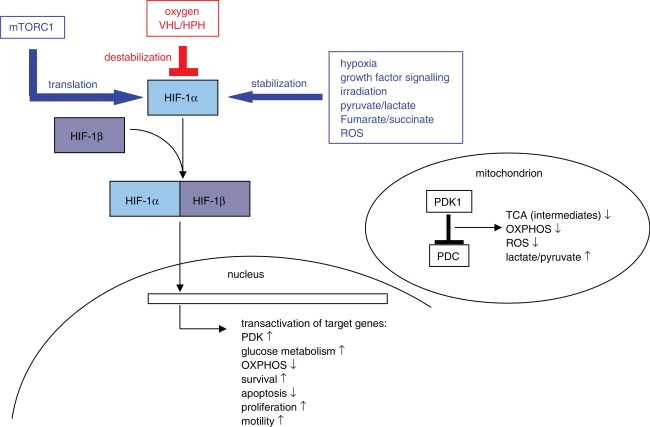
The HIF-1 network. The heterodimeric transcription factor HIF-1 consists of a β-subunit (HIF-1β), which is constitutively expressed, as well as an α-subunit (HIF-1α), the expression and stability of which is tightly regulated. Destabilizing factors for HIF-1α are indicated in red colour, whereas stabilizing factors are indicated in blue colour. Translation of HIF-1α is enhanced by the activity of mTORC1 and its downstream activities, which provides a link of HIF-1 activity to survival pathways. The proteolytic degradation of HIF-1α is mediated by the proteasome subsequent to ubiquitylation mediated by the von Hippel-Lindau (VHL)-protein. The recognition of HIF-1α is dependent on the hydroxylation of prolyl residues, which is catalyzed by HIF-1 prolyl hydroxylases 1–3 (HPH). The activity of HPH is a function of the oxygen tension within the cell. HIF-1α is stabilized by hypoxia as well as by several other factors which reflect a loss of metabolic activity in the mitochondria. HIF-1 transactivates a variety of genes which contribute to the hallmarks of aggressive cancer. Notably, at least one of the HIF-1 target genes, PDK1, directly represses the TCA and OXPHOS by the inhibition of PDC.

Intermittent hypoxia is experienced by almost every carcinoma cell and contributes to the stabilization of the heterodimeric transcription factor hypoxia-inducible factor 1 (HIF-1), which represents a key regulatory factor for the phenotypic expression of tumour cell proliferation, elevated fermentation of glucose, suppression of apoptosis, and angiogenesis (reviewed in Semenza, 2003, see Fig. 2). Whereas HIF-1β/ARNT is constitutively expressed, the HIF-1α subunit is subject to tight regulation at the translational level as well as by its stability, which is determined by the oxygen tension in the cell. HIF-1 is seen as a messenger for hypoxia which triggers transcriptional responses, the details of which are determined by the respective cellular background. However, during recent years it became increasingly clear that HIF-1 activity is not merely a response to low oxygen tension. HIF-1 was found to be evoked in response to a variety of stimuli different to hypoxia, including oncogenic signalling by ras (see above), v-src (Jiang et al. 1997), MEK-ERK (Fukuda et al. 2002), EGFR (Zhong et al. 2000), and the PI3K-AKT pathway (Zhong et al. 2000; Zundel et al. 2000; Jiang et al. 2001); but also radiation (Moeller et al. 2004), reactive oxygen species (ROS) (Brunelle et al. 2005; Guzy et al. 2005; Mansfield et al. 2005) or simply the presence of pyruvate (Lu et al. 2002) or the tricarboxylic acid cycle (TCA) intermediates fumarate (Isaacs et al. 2005) and succinate (Selak et al. 2005). In summary, activators of HIF-1 reflect a loss of metabolic activity in mitochondria, which can be caused either by defects in the respiratory chain (as suggested by Warburg, 1956), or by regulatory insults. Therefore it was not surprising, that HIF-1 was found to transactivate pyruvate dehydrogenase kinase 1 (PDK1) (Kim et al. 2006; Papandreou et al. 2006), which phosphorylates and inactivates the mitochondrial pyruvate dehydrogenase complex (PDC). In a feedback loop, this leads to a shutdown of the formation of mitochondrial acetyl-CoA and OXPHOS, which in turn substantially reduces the generation of mitochondrial ROS and contributes to the generation of lactate.

**Figure 3.**
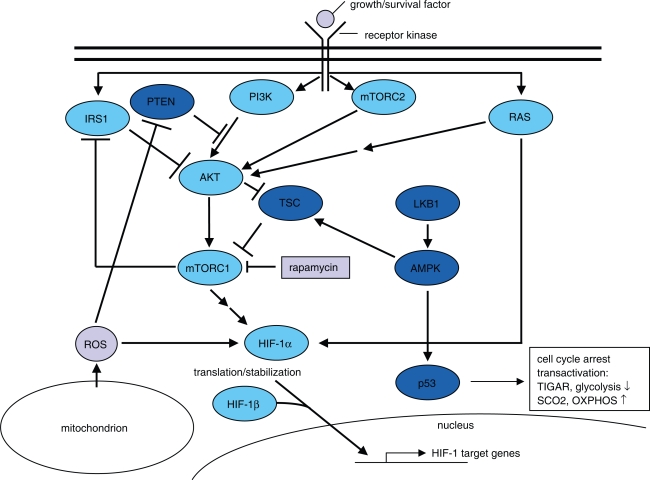
Signalling events which determine glucose utilization within the cell. The PI3K-AKT-mTOR-pathway promotes the glycolytic phenotype at various sites via the direct modulation of target proteins as well as on the transcriptional and translational level (for review see Thompson and Thompson, 2004; Sabatini, 2006). One key event supporting aerobic glycolysis is the elevated translation of HIF-1α, the effects of which are illustrated in Figure 2 and discussed in the text. Mitochondrial ROS activate the PI3K-AKT-mTOR-pathway via redox-mediated inhibition of PTEN. Active AMPK and rapamycin negatively regulate PI3K-signalling via suppression of mTORC1. Active AMPK also regulates a glucose-dependent cell cycle checkpoint via phosphorylation of p53. Intact p53 can further directly regulate both glycolysis and OXPHOS via transactivation of its target genes TIGAR and SCO2. Intracellular proteins conferring a suppressive effect on PI3K signalling are indicated in dark blue colour, whereas those which promote this pathway and enable the glycolytic phenotype are indicated in light blue colour.

Besides the supportive action of HIF-1, several studies demonstrated the significance of PI3K signalling for the Warburg effect (Fig. 3). Beneath an internal activation by e.g. ras (as described above), the PI3K pathway is activated by survival signals transmitted from outside the cell via trans-membrane receptors and mediates cell growth and survival, inhibits apoptosis, evokes angiogenesis, and activates the glycolytic phenotype (Vivanco and Sawyers, 2002; Thompson and Thompson, 2004, see Fig. 3). PI3K signalling is negatively regulated by the phosphatase and tensin homolog deleted on chromosome 10 (PTEN), a tumour suppressor which is frequently inactivated in a variety of cancers (Cully et al. 2006). PI3K signalling results in the activation of AKT and subsequently mTOR (reviewed in Thompson and Thompson, 2004; Hay, 2005). Intriguingly, PI3K signalling can be directly activated by the loss of mitochondrial respiration via a NADH-mediated inactivation of PTEN, thereby leading to elevated resistance against anticancer drugs and to a survival advantage in hypoxia (Pelicano et al. 2006b; Xu et al. 2005). This finding may explain some surprising results from mutational analyses of PTEN in different cancers, which revealed frequent hyperactivation of AKT signalling in the absence of identifiable PTEN mutations (reviewed in Cully et al. 2006). Also, this mechanism represents a feed-forward loop, by which loss of respiration may trigger a complete switch to glycolysis.

The interference of mTOR with the PI3K pathway is rather complex and probably not yet fully elucidated (reviewed in Sabatini, 2006, see Fig. 3). Two cancer relevant complexes (mTORC1 and mTORC2) have been described, which elicit distinct effects within the cell. mTORC1 is activated by PI3K signalling via AKT-mediated inhibition of its inhibitor, the tuberous sclerosis complex (TSC). mTORC1 regulates cell growth via the activation of translation and ribosome biogenesis. The complex also contributes to elevated translation of HIF-1α, which leads to stabilization of HIF-1 and the subsequent activation of several genes required for elevated glycolysis (see above).

The effects of mTORC1 are sensitive to rapamycin, analogues of which are currently under investigation for the use as anticancer agents. The mTORC2 complex, which is responsive to growth factor signalling, directly activates AKT, which leads to the confusing situation that mTOR as a protein is placed both upstream and downstream of AKT activity (Sabatini, 2006; Shaw, 2006). As mTORC1 activity leads to AKT inhibition via a feedback loop initiated through blockade of insulin receptor substrate 1 (IRS1), therapeutic application of rapamycin and its analogues may favour the oncogenic functions of activated AKT. Therefore, it is discussed to evaluate a combination of both AKT and mTORC1 inhibitors for anticancer therapy.

Thompson and colleagues demonstrated a dependence of glioblastoma cells to glycolysis following integration of constitutively activated AKT (Elstrom et al. 2004). Subsequently, pharmacologic activation of AMP activated protein kinase (AMPK), an important sensor of cellular energy tension, was found to reverse this dependency through the activation of fatty acid beta-oxidation (Buzzai et al. 2005). AMPK was recently found to be activated through phosphorylation by LKB1 (Shaw et al. 2004), which is the tumour suppressor in the rare autosomal dominant Peutz-Jeghers syndrome (PJS). This disease is characterised by the onset of gastrointestinal polyps as well as a predisposition to tumours in various other tissues (Hearle et al. 2006). Compromised AMPK activity may explain the tumourigenic phenotype resulting from LKB1 mutations, because AMPK itself exerts different tumour-suppressive functions. Via phosphorylation of the TSC1/2 complex, AMPK inactivates mTORC1 and thereby antagonizes AKT signalling (Inoki et al. 2003; Sabatini, 2006). Recently, AMPK was found to regulate a glucose dependent cell cycle arrest which is mediated by phosphorylation of the tumour suppressor p53 (Jones et al. 2005). However, p53 is inactivated in a high percentage of human cancers, and therefore this AMPK-controlled metabolic checkpoint is frequently disturbed. Interestingly, p53 was also found to modulate the balance between utilization of respiratory and glycolytic pathways by transactivating SCO2, which plays a key role in OXPHOS (Matoba et al. 2006). Another recently identified target for p53-mediated transactivation, TIGAR, was found to inhibit glycolysis by inhibiting phosphofructokinase activity (Bensaad et al. 2006). Therefore, loss of both AMPK as well as p53 function in cancer obviously contributes to the glycolytic phenotype (see Fig. 3).

In summary, both HIF-1 as well as AKT signalling can be activated by loss of respiration and contribute to elevated fermentation of glucose. AMPK signalling may confer suppressive effects on the glycolytic phenotype and cancer progression by forcing mitochondrial activity, but this requires the presence and function of other tumour suppressive mechanisms mediated by p53, PTEN, TSC1/TSC2, and LKB1 which are frequently inactivated in cancer (Jones et al. 2005; Liu et al. 2006). However, AMPK activity was also found to be involved in growth and survival of tumour models during hypoxic stress and glucose deprivation, independently of HIF-1 (Laderoute et al. 2006; Liu et al. 2006). It has been proposed, that AMPK activity may be detrimental and therefore suppressed in early cancers, while advanced cancers with inactivation of tumour suppressors may reactivate AMPK in order to acquire energy via glycolysis (Ashrafian, 2006).

In conclusion, most of the pathway deregulations described above result from cancer specific mutations and malfunctions and favour the expression of the Warburg phenotype. Many researchers suggest an enhanced rate of glucose fermentation via the Embden-Meyerhof pathway, but still there are some phenomena which are difficult to explain. In many cancers, the formation of pyruvate and acetyl-CoA are compromised by the inhibition of the respective enzymes, M2-pyruvate kinase (by formation of the low affinity dimeric form [Zwerschke et al. 1999]), and PDC (via PDK1-mediated inhibition). Concomitantly, fatty acid synthase (FASN), which catalyzes the massively observed lipogenesis in cancer cells, was suggested to represent a metabolic oncogene (Baron et al. 2004). Inhibition of this enzyme is selectively toxic to cancer cells (Lupu and Menendez, 2006). In addition, activated AKT inhibits fatty acid oxidation and contributes to fatty acid synthesis via suppression of carnitine palmitoyltransferase 1A (CPT1A) (Buzzai et al. 2005; Vankoningsloo et al. 2005; DeBerardinis et al. 2006). However, it is not clear, how the large amounts of cytosolic acetyl-CoA, which are required for the de novo synthesis of fatty acids, are generated. Hatzivassiliou et al. (2005) suggested the formation of cytosolic acetyl-CoA via citrate, which is exported from mitochondria and cleaved by ATP Citrate Lyase (ACLY), but this mechanism requires activity of the mitochondrial PDC as well as a truncated tricarboxylic acid cycle (TCA). As described above, numerous mechanisms, including PI3K and HIF-1 activity, rather repress than activate PDC. In addition, several researchers propose a substantial involvement of the PPP in the utilization of glucose in cancer cells (Boros et al. 1997; Rais et al. 1999; Cascante et al. 2000; Boros et al. 2002; Coy et al. 2005, see Fig. 1). The apparent difference between acetyl-CoA availability and utilization may be resolved by the identification and initial functional characterization of transketolase-like 1 (TKTL1), a novel enzyme with a putative key function in the fermentation of glucose within the PPP (Coy et al. 1996; Coy et al. 2005).

## TKTL1, the Pentose Phosphate Pathway, and Elevated Lipogenesis in Cancer

Although the current literature on tumour metabolism primarily focuses on the interplay between major oncogenic pathways and glucose degradation via the Embden-Meyerhof-pathway, there is evidence for a substantial involvement of the PPP in glucose metabolism in cancer cells (Fig. 1). Via its oxidative branch, the PPP delivers NADPH for reductive biosynthesis as well as for maintenance of the intracellular redox state and the detoxification of ROS. Ribose for the synthesis of nucleic acids is contributed by both the oxidative as well as the non-oxidative branch. The importance of the PPP in cancer is underlined by the efficiency of *in vivo* therapeutic approaches addressing the inhibition of this pathway (Rais et al. 1999; Ramos-Montoya et al. 2006). The anti-tumourigenic effect of the thiamine analogue and transketolase inhibitor oxythiamine (OT) was already documented two centuries ago (Trebukhina et al. 1987). Trans-ketolase represents the rate-limiting enzyme for the non-oxidative branch of the PPP, which is the main source for the generation of ribose for nucleotide synthesis within cancer cells (Boros et al. 1997; Comin-Anduix et al. 2001). Suppression of both the oxidative branch (via the G6PD-inhibitor dehydroepiandrosterone [DHEA]) as well as the non-oxidative branch of PPP (via OT) was found to exert additive effects leading to the inhibition of cancer cell proliferation (Boros et al. 1997). Ramos-Montoya et al. (2006) recently demonstrated the relevance of PPP oxidative/non-odidative balance for cancer cell survival. Combinatorial application of the inhibitors methotrexate, DHEA, and OT which address different enzyme activities necessary for the utilization of glucose for the synthesis of nucleic acids, resulted in an almost complete inhibition of cancer cell proliferation (Ramos-Montoya et al. 2006). Also, inhibition of transketolase enzyme reactions by small molecules has been demonstrated to suppress proliferation in cancer cell lines (Du et al. 2004).

Basic reactions of the non-oxidative branch of the PPP were initially delineated in the early 1950s by Horecker, Gibbs, and colleagues, who applied enzyme preparations from liver and pea tissues (Gibbs and Horecker, 1954; Horecker et al. 1954). Using acetone dry powder preparations of enzymes and radioactively labelled substrates, the scientists were able to determine reaction sequences and stoichometries in the absence of oxidative metabolism. However, although the experimental data could not be explained by the reaction scheme proposed, the latter was presented as a part of a new metabolic pathway (Horecker and Mehler, 1955). Consecutively, this pathway was commonly accepted by text-book authors. Nevertheless, until now the stoichometries of the non-oxidative PPP remained a matter of debate (reviewed in Williams et al. 1987).

The identification of TKTL1, which participates in glucose metabolism in cancer cells, sheds a new light on this pathway, as TKTL1 represents an enzyme with transketolase activity and a potentially elevated substrate spectrum with altered reaction characteristics (Coy et al. 2005). Subsequently, TKTL1 was found to be overexpressed in a wide variety of solid cancers and significantly correlated with aggressiveness in different carcinoma entities (Langbein et al. 2006; Staiger et al. 2006; Krockenberger et al. 2007; Foldi et al. 2007). TKTL1 expression was further linked to the fermentation of glucose by its elevated expression in normal tissues like testis and retina, which were already described by Warburg to perform aerobic glycolysis (Warburg et al. 1924; Coy et al. 2005). The significance of TKTL1-activity for both proliferation and glucose metabolism in cancer was recently demonstrated in functional studies using RNA interference (Zhang et al. 2007; Hu et al. 2007). Inhibition of TKTL1 was found to mediate elevated apoptosis, cell cycle arrest in the G1-phase, as well as a substantial reduction of transketolase activity. The latter finding was particularly interesting for a re-interpretation of the activity of the PPP within cancer cells. Although TKTL1 accounted for approx 50% of the total transketolase content (TKT, TKTL1, TKTL2) at the mRNA level prior to RNAi-mediated suppression, targeted knockdown of TKTL1 resulted in a >50% inhibition of total transketolase activity. As transketolase usually exists as a dimer, and the subunits cooperatively regulate the enzymatic activity of the complex (Frank et al. 2004), the latter finding may reflect a regulatory effect of TKTL1 on whole transketolase activity via heterodimerization of the enzyme with other transketolase homologues.

The classical transketolase reaction is described as the transfer of a 2 carbon unit from ketose sugars to aldose sugars (Kochetov, 1982). The reaction mechanism includes the formation of an activated glycolic aldehyde, which is subsequently transferred to the aldehyde group of the aldose acceptor substrate. Besides the 2 substrate reaction described above, TKTL1 was found to efficiently catalyze a one-substrate reaction, where the ketose sugar xylulose-5-phosphate (Xu5P) is cleaved in glycerinaldehyde-3-phosphate (GAP) and a 2 carbon product of yet unknown nature (Coy et al. 2005). This particular reactivity of TKTL1 can be explained by the loss of one exon encoding for a stretch of 38 amino acids (compared to human TKT), which contains highly conserved residues invariant for all transketolase proteins analysed so far (Coy et al. 1996). One of these residues, which corresponds to His103 in yeast transketolase, has been demonstrated to contribute to substrate specificity using the yeast enzyme (Wikner et al. 1995). Targeted mutation of His103 to alanine has been found to accelerate the one-substrate reaction, while the two substrate reaction was decelerated (Selivanov et al. 2004). Therefore, it is conceivable that TKTL1 significantly contributes to the elevated fermentation of glucose observed in tumours by the cleavage of Xu5P, a putatively irreversible activity which alters the concentration of this metabolite within the balanced equilibrium of sugars in the cytoplasm.

The balance of glucose utilization within the cell is highly regulated, and Xu5P was found to represent a messenger, the concentration of which is crucial for the coordinated regulation of the Embden-Meyerhof glycolytic pathway and the PPP (Nishimura et al. 1994; Doiron et al. 1996). In hepatocytes, Xu5P exerts its effect via the activation of Xu5P-activated protein phosphatase 2A (PP2A). This enzyme complex accelerates the Embden-Meyerhof glycolytic pathway by the dephophorylation of Fructose-6-phosphate, 2-kinase:Fructose-2,6-bisphosphatase, which itself activates phosphofructokinase by elevating the Fructose-2,6 bisphosphate levels (Nishimura and Uyeda, 1995). Furthermore, Xu5P-activated PP2A was found to induce transactivation of enzymes required for glucose-induced lipogenesis, which is mediated by dephophorylation and nuclear translocation of carbohydrate response element-binding protein (ChREBP) (Yamashita et al. 2001; Kabashima et al. 2003; Veech, 2003). Taken together, the levels of Xu5P regulate the Xu5P – PP2A—ChREBP axis for glucose utilization within the liver cell. The TKTL1 gene harbours a putative ChREBP binding site within its upstream regulatory region, which suggests a potential involvement of this axis in the regulation of TKTL1-activity. Intriguingly, Vankoningsloo et al. (2005) detected elevated ChREBP transactivation activity, glucose-induced lipogenesis, activation of PI3K-signalling, and suppression of fatty acid beta oxidation in mouse 3T3L1 pre-adipocytes after treatment with antimycin A, an inhibitor of OXPHOS (Vankoningsloo et al. 2005). These results demonstrate, that Xu5P signalling is not exclusive to cells of hepatocytic origin, but may rather represent a more ubiquitous mechanism for the regulation of glucose metabolism. Furthermore, the inactivation of OXPHOS and the resulting metabolic features in pre-adipocytes closely resemble the frequently described characteristics of aggressive cancer cells.

Given the inconsistencies in the exact stoichometries of the reactions of the PPP, the unclear origin of the large amounts of cytosolic acetyl-CoA for lipid synthesis within cancer cells, and finally the novel role of TKTL1 in glucose fermentation within cancer cells, the mechanisms leading to aerobic glycolysis need re-evaluation. It remains to be determined whether the enzymatic activity of TKTL1 contributes to glucose-induced lipogenesis, loss of OXPHOS, and the formation of lactate in aggressive cancer cells. However, TKTL1-expression correlates with this metabolism, and the cleavage of Xu5P could offer novel mechanistic perspectives for both the regulation of glucose metabolism via the Xu5P/PP2A/ChREBP axis and the origin of abundant cytosolic acetyl-CoA for lipogenesis. As both the targeted knockdown of TKTL1 expression as well as the compound-mediated inhibition of transketolase activity exerts strong inhibition of tumour growth, TKTL1 represents an excellent candidate for a targeted treatment of aggressive carcinomas.

## The Consequences of Aerobic Glycolysis: Therapy Resistance, Matrix-Degradation, Angiogenesis, and Suppression of the Immune System

It is well documented, that elevated fermentation of glucose, loss of OXPHOS, and the formation of lactate confer selective advantages to aggressive cancer cells (reviewed in Gatenby and Gillies, 2004). Although there is substantial evidence, that the generation of ROS reflects mitochondrial malfunction, either hypoxia-induced or due to mutated components of the mitochondrial electron transport chain, it is not yet clear (and most probably context-dependent) whether elevated ROS are rather advantageous or detrimental to the cancer cell (reviewed in Fruehauf and Meyskens, 2007).

Loss of OXPHOS and fermentation of large amounts of glucose has been mechanistically linked to the activation of the AKT survival pathway through the redox-mediated inactivation of PTEN (Pelicano et al. 2006b). Activation of AKT coincided with elevated resistance to several common anticancer drugs, irrespective of their intracellular targets, as well as with enhanced susceptibility to cell death induced by 3-Bromopyruvate, an inhibitor of glycolysis (Xu et al. 2005). In 2001, Gottlob et al. identified a mechanism of apoptosis suppression which is mediated by activated AKT and requires the presence of glucose, as well as the translocation of hexokinase (HK) to mitochondria (Gottlob et al. 2001). Mitochondria-targeted HK prevents apoptosis by antagonizing the release of mitochondrial cytochrome C (Majewski et al. 2004). However, whether this mechanism contributes to the enhanced resistance to anticancer agents in OXPHOS-depleted cancer cells featuring activated AKT remains to be investigated. As discussed above in more detail, also HIF-1 contributes to the phenotypic expression of different hallmarks of aggressive cancer and is associated with increased patient mortality in different cancer types (reviewed in Semenza, 2003).

At a systemic level, the elevated needs for glucose mediated by the Warburg effect may contribute to apparent alterations in cancer patients, which ultimately lead to cancer cachexia. Different studies documented an elevated glucose production in cachectic cancer patients, as well as metabolic abnormalities resembling those of type II diabetes mellitus (Holroyde et al. 1975, 1984; reviewed in Tayek, 1992). It has been suggested, that lactate production of tumours may represent a source for elevated hepatic glucose production in tumour-bearing humans (Tayek, 1992). Gluconeogenesis was found to substantially contribute to glucose production in a subgroup of cancer patients, and this metabolic activity was tightly correlated with elevated levels of serum cortisol (Tayek and Katz, 1997). Notably, several studies revealed a resistance of carcinoma cells to cytotoxic cancer therapy subsequent to the administration of glucocorticoids (GC) both *in vitro* and *in vivo* (Rieger et al. 1999; Herr et al. 2003). GC-mediated gluconeogenesis and subsequent mobilization of glucose may also further worsen the metabolic situation of the cancer patient. These data raise concerns about the frequently applied GC treatment for the suppression of cancer cachexia (Herr and Pfitzenmaier, 2006).

The formation of lactate, one of the end products of aerobic glycolysis, contributes to the acidification of the microenvironment of cancer cells. Elevated levels of intratumoural lactate were found to correlate with the likelihood of distant metastases as well as with restricted patient survival (Walenta et al. 2000). Intracellular lactate is transported across the cytoplasmic membrane by members of a family of proton-coupled monocarboxylate transporters (MCT) (Enerson and Drewes, 2003). Of these, MCT4 was found to be upregulated in a HIF-1 and PI3K-dependent manner (Moeller et al. 2005; Ullah et al. 2006), and is predominantly expressed in cells or tissues which rely on glycolysis for the generation of ATP (Dimmer et al. 2000). Once transported to the extracellular space, lactate initiates multiple activities which predominantly favour the growth and metastasis of cancers. A lactate-mediated acidification of the extracellular matrix (ECM) contributes to apoptosis in susceptible cells. However, this mechanism requires functional p53, a tumour suppressor which is frequently inactivated in advanced carcinomas (Williams et al. 1999). Therefore, aerobic glycolysis and the formation of lactate selects for the growth of (aggressive) cancer cells, which already harbour defects in tumour-suppressive pathways. The remodelling of the ECM is a prerequisite for invasive growth, and lactate contributes to this process via the modulation of fibroblasts and the activation of matrix metalloproteases (MMPs), which are required for the digestion of ECM-components. Lactate is able to stimulate the expression of the ECM-component hyaluronan (HA) and its cell surface receptor CD44 in fibroblasts (Stern et al. 2002). A HA-enriched microenvironment contributes to cancer cell survival and metastasis by promoting cancer cell migration (Zhu and Bourguignon, 2000), by enhancing anchorage-independent growth of cancer cells (Kosaki et al. 1999), and by the suppression of tumour immunogenicity (McBride and Bard, 1979) and tumour cell apoptosis (Yu et al. 1997). The acidic environment of aggressive cancer cells can promote the activation of MMPs (reviewed in Chaussain-Miller et al. 2006). The transcription of MMPs in cancer cells can be forced by CD147 (alias EMMPRIN), which is frequently expressed in human cancer (Riethdorf et al. 2006). CD147 maturation and targeting to the cytoplasmic membrane is regulated by MCT4, which provides a mechanistic link between lactate efflux and ECM remodelling (Gallagher et al. 2007). Intriguingly, CD147-expression on ovarian carcinoma cell line derived microvesicles was also demonstrated to stimulate proangiogenic activities of endothelial cells *in vitro*, suggesting a role of CD147 in tumour-angiogenesis (Millimaggi et al. 2007). By coupling the glycolytic phenotype to ECM-degradation and angiogenesis, the synergistic function of MCT4 and CD147 reflects the intimate relationship between the Warburg effect and the aggressiveness of tumours exhibiting this phenotype. In addition, lactate-mediated acidification of the tumour-microenvironment strongly suppresses proliferation and cytokine production of human cytotoxic T-lymphocytes (Fischer et al. 2007). This finding may explain the frequently observed inability of the immune system to control aggressive cancer despite of a specific T-cell response against tumour-associated antigens.

The discussion about the role of ROS in cancer is rather controversial. It has been proposed that aerobic glycolysis and the generation of mitochondrial ROS are intimately linked (Wallace, 2005; Schumacker, 2006). Also, it is commonly accepted, that ROS can damage DNA and thereby contribute to genetic instability. This might be a mechanism for cancer initiation and progression, but the over-expression of the ROS-detoxifying enzyme thioredoxin-reductase 1 (TR1) in a variety of malignant tumours rather suggests, that malignant cancers may need a delicate balance between ROS production and neutralization for oncogenic signalling, survival and resistance to therapy (Yoo et al. 2006). The neutralization of ROS by the intracellular enzyme system requires NADPH, which is mainly contributed by the PPP. In this context, it is not surprising that bacteria were demonstrated to exhibit elevated radioresistance, when glucose was predominantly metabolized by the PPP (Zhang et al. 2000). Aggressive glioblastoma cells, which frequently exhibit a high degree of resistance to antitumour therapies, revealed an increased expression of antioxidant enzymes when selected for radioresistance (Lee et al. 2004). An elevated formation of the superoxide radical via experimental inhibition of mitochondrial electron transport complex I was demonstrated to sensitize leukemia cells to anticancer agents whose action involves free radical generation (Pelicano et al. 2003). In summary, although promoting genetic instability and oncogenic signalling (by e.g. HIF-1, see above), ROS may be detrimental for the cancer cell under selection of antitumour therapy. Therefore, avoiding ROS by the elevated activity of ROS-detoxifying enzymes, shutdown of OXPHOS, as well as the utilization of PPP for the fermentation of glucose may be a suitable strategy of cancer cells for the development of resistance to radio- and chemotherapy.

## Cancer Treatment by the Suppression of the Warburg Effect

The Warburg effect is an almost universal hallmark of aggressive cancer, and since considerable evidence suggests that this particular metabolism actively contributes to cancer progression and metastasis, it is reasonable to develop anticancer strategies which target this phenotype. Indeed, experimental data suggest that the reversion of single metabolic features of the glycolytic phenotype negatively influences the aggressive properties of cancer cells. Substrate limitation as the most simple approach has been demonstrated to initiate death of cancer cells *in vitro* (Elstrom et al. 2004) and suppression of tumour growth in both mice (Zhou et al. 2007) and humans (Nebeling et al. 1995). As already discussed throughout this manuscript, an inhibition of key enzymes of affected metabolic pathways was also found to compromise growth of tumour cells (also reviewed in Pelicano et al. 2006a). Finally, there is increasing evidence that dietary components such as certain polyunsaturated fatty acids (PUFA) and plant polyphenols interfere with the glycolytic phenotype of cancer cells, and that their administration provides positive effects in the treatment of cancer.

Besides the inhibition of glycolysis and glucose-induced lipogenesis, experimental reactivation of mitochondrial OXPHOS by both genetic as well as pharmacological approaches has also been demonstrated to efficiently counteract the growth of cancer both *in vitro* and *in vivo*. Overexpression of frataxin, a protein which shows reduced expression in individuals suffering from the inherited Friedreich ataxia disease (Campuzano et al. 1997), has been shown to reduce the growth of cancer cells *in vitro* as well in a mouse xenograft model (Schulz et al. 2006). Frataxin contributes to the intramitochondrial synthesis of Fe/S clusters, which are obligate components of the respiratory chain (reviewed in Lill and Muhlenhoff, 2005). Upon overexpression, the tumour-suppressive function of frataxin was found to correlate with decreased phosphorylation of oncogenic extracellular signal-regulated kinase (ERK), increased phosphorylation and activation of the tumour suppressor p38 MAP kinase, and the reactivation of OXPHOS in different human colon cancer cell lines. In line with these results, targeted disruption of hepatic frataxin in mice was found to mediate the formation of elevated ROS, impaired phosphorylation of p38 MAP kinase, reduced activity of Fe/S cluster-containing proteins, and impaired OXPHOS in hepatocytes (Thierbach et al. 2005). As a result of these alterations, mice developed multiple hepatic tumours and had a significantly reduced life span. The importance of frataxin as an essential component of mitochondrial homeostasis clearly underlines the relevance of mitochondrial integrity for cancer suppression.

The pharmacologic reactivation of the mitochondrial PDC by dichloroacetate (DCA) was recently found to mediate profound suppressive effects on tumour growth both *in vitro* and *in vivo* (Bonnet et al. 2007). DCA inhibited PDK, which induced glucose oxidation in cancer cells. This metabolic shift coincided with a reduction of the mitochondrial membrane potential, an increase in mitochondrial ROS, and an increase in expression of the mitochondrial K^+^ channel protein Kv1.5, resulting in cancer cell apoptosis.

Although the studies on frataxin and DCA clearly show, that reactivation of OXPHOS is a promising strategy for anticancer treatment, other studies revealed differences in the reactivation capacity of tumour cell mitochondria among different cell lines. Rossignol and colleagues demonstrated a variability of substrate utilization and oxidative capacity in HeLa cells, where energy generation via forced OXPHOS correlated with an alteration of mitochondrial structure. Oxidative metabolism reduced the proliferation rate of HeLa cells, but did not result in cell death (Rossignol et al. 2004). In another study, a metabolic analysis of the non small cell lung cancer cell lines H460 and A549 revealed, that both cell lines exhibited considerable dependency on glycolysis. The extent of this dependency was different, but intimately linked to the impairment of OXPHOS, respectively (Wu et al. 2007). In HT29 colon cancer cells, the application of (oxidizable) butyrate as an energy substrate was found to initiate a differentiation process, whereas butyrate-resistant MIA pancreatic adenocarcinoma did not differentiate (Boren et al. 2003). These data may not be representative or comprehensive, but they clearly demonstrate, that there is substantial variability in the response of different cancer cells on the modulation of OXPHOS. The responses include reduced proliferation, cell cycle arrest, cell death, and differentiation. To which extent these responses contribute to a therapeutic success *in vivo* remains to be investigated. The intracellular balance of ROS elimination and formation, the latter of which may reflect the degree of damage in the mitochondrial electron transport chain, may play a major role in the cellular response to forced OXPHOS. In conclusion, substrate limitation or specific inhibition of glucose fermentation may represent a more comprehensive therapeutic option, because these approaches do not aim at the reactivation of a tumour-suppressive process, but instead target the essential (“oncogenic”) process of energy generation in cancer cells.

Certain dietary components, which have been identified to enhance the positive effects of chemotherapy, may also interfere with the Warburg effect. The application of omega-3 (n-3) fatty acids was repeatedly demonstrated to enhance the tumour cytotoxic effects of chemotherapy, to improve the quality of life during therapy, while reducing cancer cachexia (reviewed in Hardman, 2004). PUFA, including n-6 and n-3 fatty acids, were recently found to suppress glycolytic and lipogenic gene expression in a ChREBP-dependent manner (Dentin et al. 2005). Whether this mechanism contributes to the observed beneficial effects of n-3 fatty acids by modulation of the Warburg effect in cancer cells remains to be investigated.

Several plant polyphenols, which are also frequently categorized as antioxidants, were found to exert beneficial effects in the treatment of cancer, predominantly by a sensitization of tumour cells to chemo- and radiotherapy (reviewed in Garg et al. 2005). The application of resveratrol, a constituent of red wine, induced cell cycle arrest in lymphoma cells, which coincided with the inhibition of PI3K-mediated glucose metabolism (Faber et al. 2006). Resveratrol also stimulates the PGC-1α-mediated expression of genes required for OXPHOS by enhancing the affinity of NAD+ to the histone deacetylase Sirt1 (Lagouge et al. 2006). In this context, the application of resveratrol mimics the effects of physical exercise or fasting, resulting in an improvement of mitochondrial function and elevated OXPHOS. This mechanism may contribute to the anti-tumourigenic effects of resveratrol, and there is evidence, that other plant polyphenols, e.g. curcumin, also interfere with mitochondrial activity and the generation of ROS (Garg et al. 2005; Su et al. 2006). Recently, a combined application of plant polyphenols and PUFA within a standardized study for advanced cancer patients with cancer anorexia/cachexia and oxidative stress revealed safety and efficiency with regard to the improvement of clinical, nutritional, and laboratory features as well as the quality of life (Mantovani et al. 2006)

In summary, both the inhibition of glucose fermentation (via specific inhibition of enzymes and/or dietary intervention) as well as the reactivation of OXPHOS exert a tumour-suppressive effect on cancer cells, which should be further investigated for the development of novel therapeutic strategies. Within this cellular response, the role of ROS is probably important, but a causative involvement of ROS remains enigmatic and requires further analysis. In mice, the dietary substrate limitation by the application of a ketogenic diet in a low-caloric dosage reduced tumour growth (Zhou et al. 2007), which suggests an antitumour effect that is mainly based on caloric restriction. However, to prevent cancer-cachexia, we propose the application of a carbohydrate-restricted nutrition, which includes significant amounts of (n-3) fatty acids as well as selected plant polyphenols. Considering the observations discussed above, this strategy could represent a novel and promising approach for a diet-based intervention against aggressive cancer.

## Conclusions

It has been experimentally demonstrated, that the inhibition of glucose fermentation, substrate limitation, inhibition of lactate generation, inhibition of glucose-induced lipogenesis, as well as the reactivation of OXPHOS represent promising strategies for cancer therapy. Therefore it seems, that all of the features which characterize the metabolism of aggressive cancer, are essential for its phenotype. Clinically, the altered glucose metabolism in cancer patients is often reflected by apparent systemic metabolic abnormalities, which resemble those seen in type II diabetes mellitus and frequently result in cancer cachexia (Tayek, 1992). Since both metastatic growth of cancer cells as well as cachexia represent major causes of cancer-related death, the investigation of pharmacological and diet-based approaches against glucose fermentation of tumours is of utmost importance.

TKTL1, one of three human transketolase homologues, most likely plays a key role in aerobic glycolysis. Besides a positive correlation of TKTL1-expression, aerobic glycolysis, and the aggressiveness of different cancer types, targeted knockdown of TKTL1 *in vitro* already demonstrated its functional relevance for both growth and metabolism of cancer cells (Coy et al. 2005; Langbein et al. 2006; Krockenberger et al. 2007; Zhang et al. 2007; Hu et al. 2007).

Given the novel role of TKTL1 in the metabolism of aggressive cancer cells, as discussed within this article, the pathways of glucose fermentation require a thorough re-evaluation. The glycolytic phenotype enables cell survival by a rapid utilization of large amounts of glucose in the absence of OXPHOS. This metabolism may support a proliferative phenotype in the absence of ROS in both cancer and normal cells (Brand and Hermfisse, 1997). Notably, recent studies in lymphocytes suggest a role of PI3K signalling in the glycolytic control of cell growth, where entry into the S-phase of the cell cycle is preceded by a shift to lactate production via the PPP (Doughty et al. 2006). Utilization of the PPP may reflect the cellular demand for ROS-free glucose-derived energy during replication and subsequent cell division, since genomic DNA is temporarily unprotected in these phases. It has been demonstrated, that elevated ROS during replication may provoke telomere loss leading to the inhibition of proliferation and cellular senescence (von Zqlinicki et al. 1995, Matthews et al. 2006). During cell division, compartmentalization is partially abolished. In this situation, cellular respiration would release ROS into the same compartment where genomic DNA is located, which could cause severe DNA mutations. Otherwise, a mitochondria-independent energy production via TKTL1-mediated fermentation of glucose within the PPP would minimize ROS release.

Consistent with the proposed role of TKTL1 in energy generation during the cell cycle, Warburg already described that aerobic glycolysis is not restricted to cancer, but also occurs in healthy tissues like e.g. testis and retina (Warburg et al. 1924). Besides in these two tissues, the TKTL1 protein is also strongly expressed in endothelial and neuronal cells (Coy et al. 2005). In testicular tissue, male germ cells exhibit a high proliferative rate, and DNA of these cells must be protected against ROS induced mutations during replication, which may be supported by a fermentative glucose metabolism mediated by TKTL1. A similar strategy, favouring rapid cell growth and avoiding DNA mutations, may be applied by cancer cells which exploit the fermentative glucose metabolism for energy production. The protective effect of the fermentative energy production is most probably based on an ATP production without the formation of mitochondrial ROS, as well as on the efficient production of NADPH (and concomitantly reduced glutathione) via the oxidative part of the PPP. As a consequence, cancer cells with metabolically active mitochondria (OXPHOS) are more sensitive against standard cancer therapies, whereas cancer cells with a fermentative energy production are resistant against most standard chemotherapies (Pelicano et al. 2003; Xu et al. 2005). In retina, endothelia, and neuronal cells, a major function of TKTL1 could be the rapid elimination of large amounts of glucose. Thereby, TKTL1 may prevent the formation of toxic glucose adducts (advanced glycation end products, AGE), which are frequently seen in diabetic lesions and neurodegenerative diseases. This suggestion lends support from the finding, that the administration of benfotiamine (an analogon of the cofactor thiamine and transketolase activator) blocks hyperglycaemic damage and prevents experimental diabetic retinopathy in mice (Hammes et al. 2003). We propose, that TKTL1 mediates aerobic glycolysis, which prevents hyperglycaemic damage as well as the formation of ROS, and which may be a prerequisite for rapid cell growth as observed in both male germ cells and cancer. Therefore, the enzymatic function of TKTL1 seems Janus-faced, exerting both protective as well as destructive effects for the organism. In the case of cancer, the switch from OXPHOS to a predominant/obligatory fermentative metabolism seems to occur with differential efficiency within different tissues, and this might be reflected by the respective frequencies of aggressive tumours. Whereas epithelial tissues are frequently affected from metastatic cancer, mesenchymal tissues, e.g. the heart muscle, very rarely develop aggressive neoplasia. This may be caused by the preference of these tissues for an oxidative metabolism. Even in the presence of glucose, the heart muscle preferentially utilizes fatty acids, ketone bodies, or lactate for an oxidative generation of ATP. Interestingly, the heart muscle strongly expresses a short splice variant of TKTL1, which putatively encodes for a N-terminally truncated isoform of the protein (Coy et al. 1996).

The modern western lifestyle is characterized by a dramatic decrease in physical activities as well as by the ingestion of food displaying a high glycaemic index, which results in an intermittent fast and strong increase in blood glucose level, massive secretion of insulin, and extensive glycogen storage. In parallel, the incidence of pathological metabolic conditions such as obesity, type 2 diabetes, and the metabolic syndrome is rising continuously. These metabolic disturbances are associated with increased incidence and/or mortality for a number of cancers (Giovannucci and Michaud, 2007). We propose, that the protective capacity of the TKTL1-pathway is not sufficient to compete with a chronic glycaemic overload, a condition which favours the development of several civilization-related diseases like the Alzheimer disease, type 2 diabetes, and the metabolic syndrome. On the other hand, the elevated availability of glucose predisposes cancerous cells to switch from OXPHOS to a fermentative metabolism in both the absence and presence of oxygen, which favours an aggressive phenotype. The latter suggestion is further supported by studies which link physical activities (and thereby the reduction of glycogen reserves) to elevated cancer survival in humans (Demark-Wahnefried, 2006) and attenuated tumour growth in animals (Michna et al. 2006; Colbert et al. 2006). In line with this idea, human populations which traditionally ingest a low-carbohydrate diet which is combined with high amounts of protein and PUFA, reveal a comparatively low incidence of aggressive cancer (Stefansson, 1960). Also, domestic animals (e.g. cats and dogs) which usually consume western diets with a comparatively high glycaemic index, frequently suffer from aggressive cancer, whereas carnivore animals and herbivore animals do have a low rate of metastasizing cancer and rarely die from this disease. Both carnivores and herbivores predominantly live from proteins and fat/oil. Although herbivores ingest large amounts of complex carbohydrates (cellulose and other fibres), these are fermented to fatty acids by bacteria within the gastrointestinal tract and therefore exhibit an extremely low glycaemic index. Both the herbivore as well as the carnivore type of diet are well established during evolution, and due to the low glycaemic index, both do not further result in the secretion of excessive amounts of insulin. Since glucose represents the substrate necessary for a switch from an OXPHOS-mediated ATP production to a fermentative ATP production, limited release or even absence of glucose during digestion may explain the low rates of cancer-caused mortality in herbivore and carnivore animals.

Otto Warburg reasoned, that a fermentative metabolism of glucose would be a more ancient way for the generation of energy, which is less susceptible to disturbances mediated by gene mutations, as e.g. in the case of cancer. In contrast, the mitochondria-based energy production is highly efficient, but very sensitive to inactivating events. DNA of mitochondria, organelles originating from bacteria which have been integrated during evolution, is more sensitive to mutations than nuclear DNA due to reduced repair mechanisms and a higher density of coding DNA. Mitochondrial DNA mutations, unspecific antibiotics which also inhibit mitochondria, as well as pesticids and heavy metals, which in part inactivate mitochondria, can favour a shift from a mitochondria-based energy production to energy production by glucose fermentation, which is less susceptible to mutational damage. For affected cells, this shift may be further facilitated by an evolutionary novel microenvironment within the organism itself, which is characterized by a permanent availability of high amounts of glucose due to a nutrition with a high glycaemic index, the absence of periods of starvation, as well as reduced physical activity.

It remains to be determined, whether TKTL1 plays a key role in the fermentative metabolism observed in the tissues, where the enzyme is strongly expressed. However, we propose that the high rate of carbohydrate ingestion may contribute to various metabolic diseases, including the development of aggressive cancer. We further suggest that both dietary intervention as well as targeted modulation of TKTL1 glucose metabolism may be promising approaches for anticancer therapy as well as the treatment of other civilization-related diseases.

## Abbreviations Used

OXPHOS: oxidative phosphorylation;
FDG-PET: [^18^F] fluoro-2-deoxyglucose positron emission tomography;
PPP: pentose phosphate pathway;
RNAi: RNA interference;
ROS: reactive oxygen species;
PDC: pyruvate dehydrogenase complex;
PJS: Peutz-Jeghers syndrome;
TCA: tricarboxylic acid cycle;
OT: oxythiamine;
DHEA: dehydroepi-androsterone;
Xu5P: Xylulose-5-phosphate;
GAP: glycerinaldehyde-3-phosphate;
DHAP: dihydroxyacetone-phosphate;
ECM: extracellular matrix;
HA: hyaluronan, hyaluronic acid;
MMP: matrix metalloprotease;
MCT: monocarboxylate transporter, PUFA, polyunsaturated fatty acid;
DCA: dichloroacetate;
AGE: advanced glycation end product.
